# Beneficial Effects of Spore-Forming *Bacillus* Probiotic Bacteria Isolated From Poultry Microbiota on Broilers' Health, Growth Performance, and Immune System

**DOI:** 10.3389/fvets.2022.877360

**Published:** 2022-05-31

**Authors:** Maria S. Mazanko, Igor V. Popov, Evgeniya V. Prazdnova, Aleksandr G. Refeld, Anzhelica B. Bren, Galina A. Zelenkova, Vladimir A. Chistyakov, Ammar Algburi, Richard M. Weeks, Alexey M. Ermakov, Michael L. Chikindas

**Affiliations:** ^1^Center for Agrobiotechnology, Don State Technical University, Rostov-on-Don, Russia; ^2^Academy of Biology and Biotechnology, Southern Federal University, Rostov-on-Don, Russia; ^3^ChemBio Cluster, ITMO University, Saint Petersburg, Russia; ^4^Department of Biotechnology, College of Science, University of Diyala, Baqubah, Iraq; ^5^Health Promoting Naturals Laboratory, School of Environmental and Biological Sciences, Rutgers State University, Bridgeton, NJ, United States; ^6^Department of General Hygiene, I.M. Sechenov First Moscow State Medical University, Moscow, Russia

**Keywords:** *Bacillus*, probiotics, poultry, growth performance, IL-6, IL-10, gut microbiota, spore germination

## Abstract

Probiotics are known for their beneficial effects on poultry health and wellbeing. One promising strategy for discovering *Bacillus* probiotics is selecting strains from the microbiota of healthy chickens and subsequent screening for potential biological activity. In this study, we focused on three probiotic strains isolated from the gastrointestinal tract of chickens bred in different housing types. In addition to the previously reported poultry probiotic *Bacillus subtilis* KATMIRA1933, three strains with antimutagenic and antioxidant properties *Bacillus subtilis* KB16, *Bacillus subtilis* KB41, and *Bacillus amyloliquefaciens* KB54, were investigated. Their potential effects on broiler health, growth performance, and the immune system were evaluated *in vivo*. Two hundred newly hatched Cobb500 broiler chickens were randomly divided into five groups (*n* = 40). Four groups received a standard diet supplemented with the studied bacilli for 42 days, and one group with no supplements was used as a control. Our data showed that all probiotics except *Bacillus subtilis* KATMIRA1933 colonized the intestines. Treatment with *Bacillus subtilis* KB54 showed a significant improvement in growth performance compared to other treated groups. When *Bacillus subtilis* KB41 and *Bacillus amyloliquefaciens* KB54 were applied, the most significant immune modulation was noticed through the promotion of IL-6 and IL-10. We concluded that *Bacillus subtilis* KB54 supplementation had the largest positive impact on broilers' health and growth performance.

## Introduction

Spore-forming probiotics that are beneficial for animals have been reported as being isolated from soil (1), aquatic systems (2), and other sources, such as fermented dairy products (3). It has been reported that the spores of many *Bacillus* strains germinate and proliferate in the intestine of animals (4–8) and can sporulate in the lower intestine (9, 10). Experiments with a simulated gastrointestinal tract model showed that cells under these conditions are metabolically active (11, 12).

Some authors suggest that spore-forming probiotics, especially *Bacillus* species, have advantages over other probiotics due to their encapsulation ability which is associated with their survival and colonization in the digestive tract (13, 14). The beneficial effects of *Bacillus* probiotics in the poultry industry include improving (i) egg quality and production rate, (ii) body weight (BW), (iii) average daily weight gain (ADWG), (iv) feed intake (FI), (v) feed conversion ratio (FCR) and (vi) meat and sperm quality (14–19). However, some studied *Bacillus* probiotics did not positively impact the above-mentioned parameters (14, 20, 21). Therefore, qualitative studies should be conducted to investigate the beneficial influence of spore-forming probiotics on the poultry industry (14).

Our study used three potential probiotic bacilli strains isolated from chicken feces: *B. subtilis* KB16, *B. subtilis* KB41, and *B. amyloliquefaciens* KB54. *B. subtilis* KATMIRA1933 was isolated from a dairy product and was used as a reference probiotic strain (3). The probiotic potential of the tested bacilli was evaluated for growth performance, gastrointestinal colonization, and immune modulation of cage housed Cobb500 broiler chickens.

## Materials and Methods

### Probiotic Strains and Probiotics Preparation

For this study, bacilli with antioxidant and antimutagenic properties isolated from chickens' feces were used. Fecal samples were collected from three cage poultry farms, a one-floor poultry farm, and three free-range poultry farms in the Rostov and Krasnodar regions of the Russian Federation. For free-range chickens, fecal samples were only taken from portions of the feces that were not in direct contact with the soil. Samples were collected in sterile containers and were transferred to the laboratory within 24 h.

Luria-Bertani (LB) medium was used to isolate intestinal bacilli. Bacilli were selected among other bacteria according to the morphology of the colonies. This was further confirmed using microscopy for the selection of spore-forming rods (PrimoStar, Zeiss, Jena, Germany). Individual strains were identified by MALDI-TOF mass spectrometry at the Rostov Research Institute of Microbiology and Parasitology, Rostov-on-Don. The study was performed on a Microflex LT instrument (Bruker Daltonics GmbH, Leipzig, Germany) using Biotyper (version 3.0) software (Bruker Daltonics GmbH). Overall, nineteen bacilli strains were selected from the litter.

To determine the safety of the selected strains, each was assessed for several potentially harmful properties, including antibiotic resistance. To determine hemolytic activity, all strains were plated on blood agar and incubated at 42°C for 48 h. To determine the sensitivity to antibiotics, the strains were seeded on appropriate solid nutrient media to a final concentration of 10^6^ CFU/g. Next, standard antibiotic disks (HiMedia, Mumbai, India) were placed onto the inoculated media. Amoxicillin (30 μg), ciprofloxacin (5 μg), ceftriaxone (30 μg), azithromycin (30 μg), erythromycin (15 μg), tetracycline (30 μg), and gentamicin (10 μg) (all HiMedia) were used, with 4 disks per plate with 2 replicates. The results were determined after 48 h of incubation at 42°C. Bacterial biosensors were used to estimate the DNA-protective and antioxidant activities of bacterial fermentates. We used *E.coli* MG 1655 carrying a plasmid with luminescence genes controlled by a stress-inducible promoter as biosensors. *E.coli* MG 1655 pRecA-lux reacts to DNA damage and *E. coli* MG1655 pKatG-lux reacts to oxidative stress. The details of this method are described in Zavilgelsky et al. and Prazdnova et al. (22, 23).

After completing the above-mentioned experiments, three strains were selected for feed supplementation as potential probiotics: *B. subtilis* KB16, *B. subtilis* KB41, and *B. amyloliquefaciens* KB54. The fermented milk product isolate, *B. subtilis* KATMIRA1933 [tested in our previous studies (24–28)], was obtained from the Russian National Collection of Industrial Microorganisms (RNCIM, Moscow, Russia) and used as a positive control.

The feed additives for these strains were prepared using solid-phase fermentation (29). Briefly, soybeans were inoculated with an overnight culture of the studied *Bacillus* strains and grown for 2 days at 42 °C. The fermented substrate was then milled and dried. The milling equipment used to prepare the solid-phase materials was sanitized with 95% ethanol. The spore content of each feed additive was 10^8^ CFU/g with a final spore count of 10^5^ CFU/g in the poultry feed.

### Experimental Design

A total of 200 newly hatched Cobb 500 broiler chickens were randomly divided into five groups, including the control group, with four replicates per group and ten broilers for each replicate. The control group was fed without probiotic supplementation to the diet (CON). The four treatment groups were labeled as T1, T2, T3, and T4, and their feed was supplemented with *B. subtilis* KATMIRA1933 (0.1%), *B. subtilis* KB16 (0.1%), *B. subtilis* KB41 (0.1%), and with *B. amyloliquefaciens* KB54 (0.1%), respectively.

### Birds, Diet, and Management

This study was carried out for 42-day at the Center for Agrobiotechnology of Don State Technical University. Ten birds per cage were housed under the following light cycles: 24 h light for day 1, 23 h light/1 h dark for day 2, 18 h light/6 h dark for days 3–9, 15 h light/9 h dark for days 10–20, 12 h light/12 h dark for days 21–35, 23 h light/1 h dark for days 36–42. Cage sizes were: height – 50 cm, width - 95 cm, and depth - 70 cm. The planting density per bird was - 0.066 m^2^. There was one vacuum drinker with a volume of 3 liters per cage. The feeding front was 9 cm with a total length of the feeder of 90 cm. The initial room temperature was fixed at 32°C on day 1 and gradually lowered until it reached 21°C on day 21. For days 22–42, the temperature was constantly held at 21°C. The air humidity was 60–65% for the duration of the experiment. The temperature and humidity regimens were maintained throughout the poultry house. Ventilation in the poultry house was natural supply and exhaust. The compound feed manufactured by the “BEST” company was used for feeding during three phases: STARTER (days 1–14), GROWER (days 15–28), and FINISHER (days 29–42) (Table 1). The feed and feeding of the animals corresponded to the GOST P 51899-2002 “Granulated mixed feeds. General specifications”^1^ The feed compositions for all three phases included wheat, corn, soybean meal, wheat gluten, corn gluten, corn cake, soybean cake, sunflower cake, sunflower oil, tricalcium phosphate, lysine, methionine, threonine, protein-vitamin-mineral concentrates. The temperature, humidity, and light conditions were constant in all groups. Water and feed were provided *ad libitum* during the 42-day study period. No lethal cases were observed.

**Table 1:** Nutrient content of the experimental diets^a^.

**Items**	**Feeding phase**		
	**Starter**	**Grower**	**Finisher**
**100 g of compound feed contains:**			
Metabolic energy, kcal	312.00	318.00	326.00
Crude protein,%	24	22.75	19.05
Crude fat,%	5.82	6.91	6.06
Linoleic acid, %	2.79	2.53	2.74
Crude fiber,%	4.1	4.08	4.40
Crude ash, %	0	0	4.52
Lysine, %	1.43	1.24	1.09
Methionine, %	0.72	0.60	0.58
Methionine + cystine, %	1.07	0.93	0.86
Threonine, %	0.94	0.87	0.74
Tryptophan, %	0.31	0.29	0.22
Ca, %	1.10	1.00	1.04
P (absorbable), %	0.59	0.54	0.57
Na, %	0.17	0.16	0.18
**1000 g of compound feed contains:**			
Vitamin A, 1000 IU	12000.00	11000.00	11000.00
Vitamin D3, 1000 IU	5000.00	5000.0	4000.00
Vitamin E, mg	110.00	75.00	70.00
Vitamin K, mg	0	0	2.00
Vitamin K3, mg	3.00	4.00	0
Vitamin B1, mg	3.00	3.00	20.00
Vitamin B2, mg	9.00	8.00	5.00
Vitamin B3, mg	15.00	18.00	18.00
Vitamin B4, mg	60.00	60.00	0
Vitamin B5, mg	60.00	4.00	35.00
Vitamin B6, mg	6.00	0.02	4.00
Vitamin B9, mg	2.00	30.00	1.50
Vitamin B12, mg	2.00	1.75	0.01
Vitamin C, mg	0	30.00	30.00
Vitamin H (biotin), mg	0.20	0.20	0.10
Mo, mg	0	1.00	1.00
Fe, mg	80.00	80.00	80.00
Cu, mg	12.00	8.00	8.00
Zn, mg	150.00	80.00	80.00
Mn, mg	100.00	100.00	100.00
Co, mg	0	1.00	1.00
I, mg	1.00	1.00	1.00
Se, mg	0.40	0.25	0.25
*B. subtilis* KATMIRA1933, %	0.1 (T1) 0 (T2, T3, T4)	0.1 (T1) 0 (T2, T3, T4)	0.1 (T1) 0 (T2, T3, T4)
*B. subtilis* KB16, %	0.1 (T2) 0 (T1, T3, T4)	0.1 (T2) 0 (T1, T3, T4)	0.1 (T2) 0 (T1, T3, T4)
*B. subtilis* KB41, %	0.1 (T3) 0 (T1, T2, T4)	0.1 (T3) 0 (T1, T2, T4)	0.1 (T3) 0 (T1, T2, T4)
*B. amyloliquefaciens* KB54, %	0.1 (T4) 0 (T1, T2, T3)	0.1 (T4) 0 (T1, T2, T3)	0.1 (T4) 0 (T1, T2, T3)

^a^*https://best-korm.ru/indicators-broiler*.

#### Samples

At the end of the study, all birds were sacrificed. Blood samples were collected from the axillar vein of two randomly chosen birds per replicate into two vacuum tubes (10 mL) per bird containing coagulant. The collected samples were aliquoted for use in several different assays. They were used to analyze leukocyte composition *via* counting leukocytes in blood smears stained with Diachim-Diff-Quik staining (Agath-Med, Moscow, Russia) using microscopic morphometry. After centrifugation (3,000 × g for 10 min), serum samples were examined for total protein, albumin, albumin/globulin ratio, glucose, and cholesterol levels using an automatic biochemistry analyzer A-15 (BioSystems, Barcelona, Spain).

Before sacrificing, the birds were not fed for 10 h according to the GOST 18292-2012 “Slaughter poultry. Specifications”^2^ The gut of two randomly chosen birds per replicate was ligated and removed from the carcass. The gut content of each gastrointestinal section was collected into sterilized containers and immediately kept at 4°C for further assays. Bacterial analysis was carried out within 24 h. Spleens were aseptically collected into sterilized containers and immediately kept at −80°C for further experiments.

#### Measurement of Growth Performance

Mean replicate feed intake (FI), and each chicken's body weight (BW) were measured at 7, 14, 21, 28, 35, and 42 days of age. Average daily weight gain (ADWG) was calculated from obtained data according to the following formula:


$$\begin{array}{l} \text{ADWG}=\frac{\text{Initial BW }\left(\text{g}\right)-\text{ Final BW}\left(\text{g}\right)}{\text{Time interval }\left(\text{days}\right)} \end{array}$$

Feed conversion ratio (FCR) was calculated as the mean replicate FI (g) per BW of each bird (g).

### Bacterial Analysis

#### Bacterial Isolation

The small intestine and cecum contents were plated on solid nutrient media. The appropriate selective media was used to determine the numbers of lactic acid bacteria (LAB) (MRS, LenReactiv), *Bifidobacterium* (Bifidobacterium Broth, HiMedia), *Enterococcus* (Enterococcus Confirmatory Agar, HiMedia), *Escherichia coli*, and lactose-positive bacteria (Endo Agar, HiMedia).

#### Survival of Bacterial Strains in the Intestine

Vegetative cells and spores of the studied bacilli were enumerated, following the methods described by Watterson et al. (30) with some modifications. LB medium was used to propagate bacilli. The total number of bacilli (N_all_), including both vegetative cells and spores, was detected by plating on LB agar. Bacilli colonies, among other bacterial species, were observed morphologically. Since the number of different colony morphoforms was small, each morphoform was confirmed by microscopy (PrimoStar, Zeiss) as a spore-forming rod. The number of spore forms (NS) in the LB medium was determined after the pasteurization of the suspension at 95°C for 5 min. Specifically, the sample was divided into two aliquots. The first aliquot was immediately pasteurized (1 min. at 95°C to kill all vegetative cells) and plated on LB. The survived bacilli grew from the germinated spores present in the original sample. The second aliquot was incubated at +4°C for 24 h to induce sporulation in the spore-forming bacilli. Our previous study (data not published) indicates that almost 100% of the bacilli we are working with are sporulating after being exposed to cold stress. Then, the sample was pasteurized and plated to enumerate the spore-forming bacilli germinated from the formed spores. In this case, the numbers were representing those present as spores in the original sample and those sporulated after exposure to the cold stress (“original spores” + “original vegetative cells”). By subtracting the aliquot #1 numbers from the aliquot #2 numbers we were getting the number of vegetative cells of sporeforming bacilli in the original sample. Also, by plating the non-pasteurized sample we were getting the total numbers of the microorganisms in the original sample, from which the numbers of non-sporeforming microorganisms was easy to derive. Therefore, the number of vegetative forms was observed as:


$$\begin{array}{l} N_{V}\;=\;N_{all}-N_{S}. \end{array}$$

### IL-6 and IL-10 Gene Expression Analysis

Total RNA was isolated from the spleen of one chicken for each replicate with four chickens in total per group (31). Isolation of total RNA from the samples was carried out by the phenol-chloroform method using the ExtractRNA reagent (Evrogen, Moscow, Russia). Spleen samples (100 mg) were homogenized in a mortar with liquid nitrogen. Total RNA was purified using the CleanRNA Standard kit (Evrogen) following the provided protocol. All actions were carried out following the instructions for the corresponding kits from the manufacturer. Primers targeting IL-10, IL-6, and reference (β-actin) genes were previously described in the literature (32).

The reverse transcription reaction was carried out using the MMLV RT kit protocol (Evrogen). qPCR from the obtained cDNA was performed using the qPCRmix-HS SYBR kit (Evrogen) on a Bio-Rad CFX96 amplifier. Experiments were conducted following the protocols of the kit manufacturers. The results were analyzed using the Bio-Rad CFX Manager software (Hercules, CA, USA).

Expression levels were normalized to β-actin, which was used as a reference gene. The change in the expression level of the target genes was calculated using the ΔΔCt method as a fold change in gene expression in experimental samples relative to the control sample. The difference was considered statistically significant at p < 0.00625, taking the Bonferroni correction into account (33).

### Statistical Analysis

IBM SPSS 26.0 (SPSS, Inc., Chicago, IL, USA) was used for data analysis. Data were not normally distributed according to the Shapiro–Wilk test. The Kruskal-Wallis test, followed by the Dunn-Bonferroni *post-hoc* test, was used to evaluate different medians among treatments. Results were presented as mean ± standard deviation. The statistical significance was determined as *p* < 0.05.

### Ethical Statement

The study was approved by the Ethics Committee of the Don State Technical University, Rostov-on-Don, Russia (Protocol No. 67-43-2).

## Results

### *In vitro* Properties of Isolated Bacilli

All nineteen selected strains were examined for safety. Hemolytic activity, mutagenicity (by lux-biosensors), and antibiotic resistance were examined. None of the strains were promutagenic, hemolytic, or resistant to the tested antibiotics, however, some of them showed prooxidant activity.

**Figure 1:**
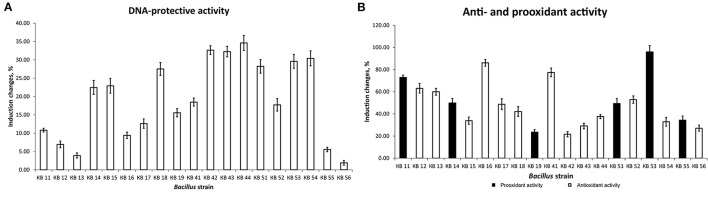
DNA-protective **(A)** and anti- and prooxidant **(B)** effects of *Bacillus* strains.

The strains were examined for antioxidant and antimutagenic activity using lux-biosensors. Figure 1 demonstrates the difference in the properties of *Bacillus* strains. For further studies, three strains out of nineteen isolated bacilli with the highest pooled antimutagenic and antioxidant activities were selected, which were *B. subtilis* KB, *B. subtilis* KB41, and *B. amyloliquefaciens* KB54.

### Leukocyte Composition and Biochemical Blood Analysis

**Figure 2:**
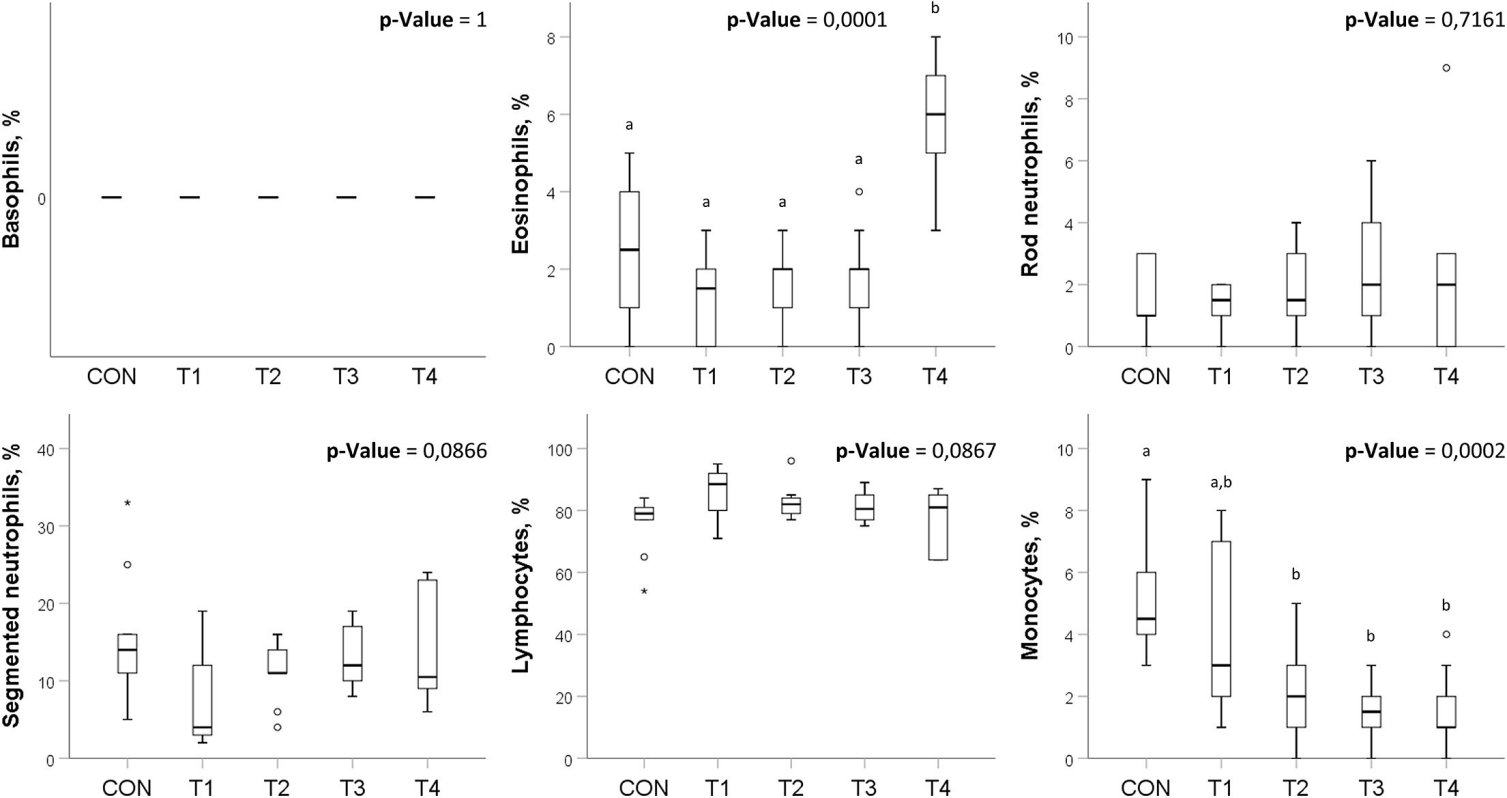
Leukocyte formula presented as boxplots in broilers treated with probiotics. Means within the same row without common superscripts are significantly different (*p* < 0.05). CON, control group; T1, group treated with *B. subtilis* KATMIRA1933; T2, group treated with *B. subtilis* KB16; T3, group treated with *B. subtilis* KB41; T4, group treated with *B. amyloliquefaciens* KB54. The data was obtained from two birds per replicated with 8 birds per group in total.

There were no significant differences in basophils, rod neutrophils, segmented neutrophils, and lymphocytes counts among the groups. However, there was a significant increase in eosinophils in the T4 group and a significant decrease in monocytes in the T2, T3, and T4 groups (Figure 2).

**Figure 3:**
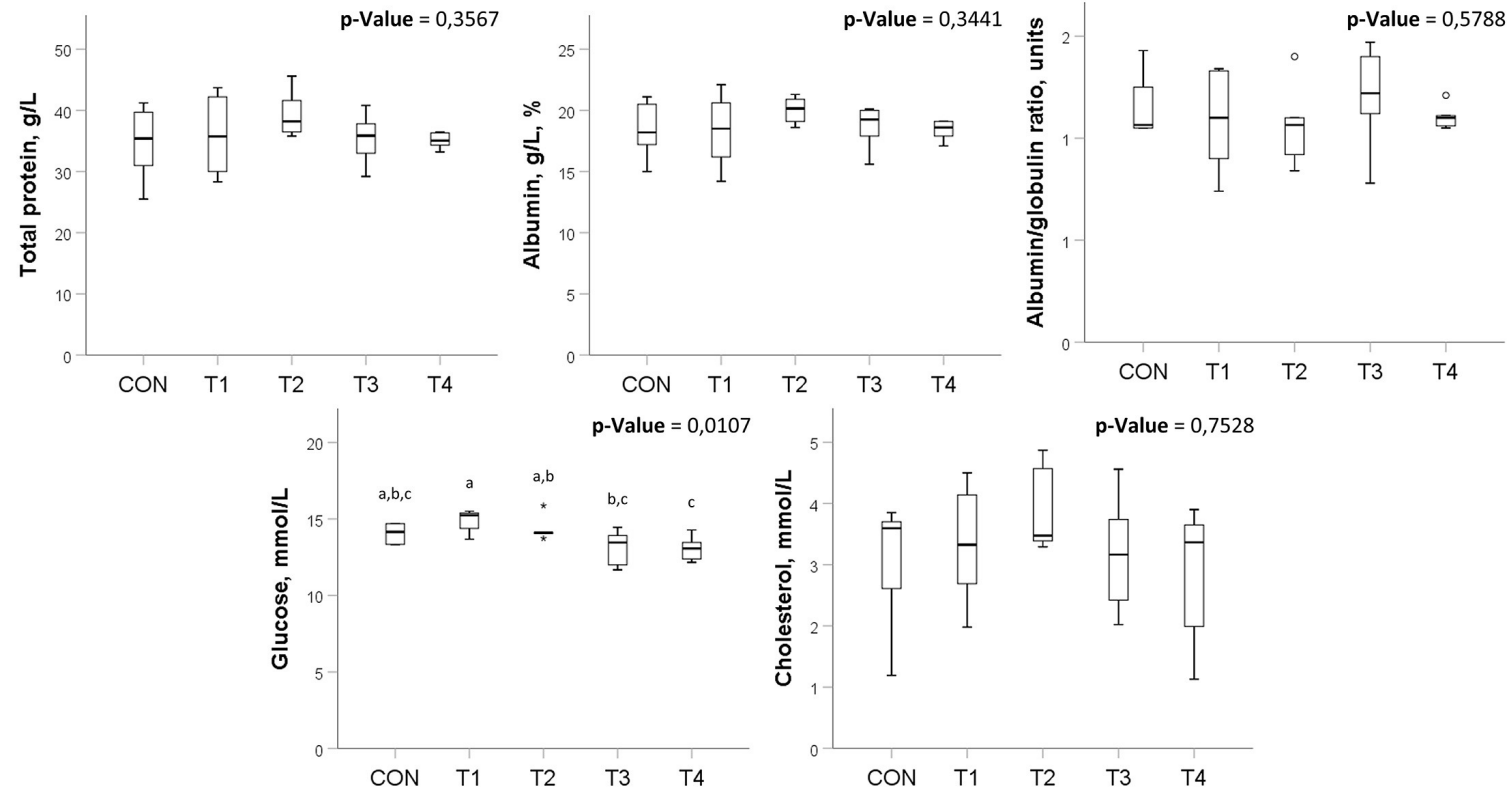
Biochemical blood analysis in broilers treated with probiotics. Means within the same row without common superscripts are significantly different (*p* < 0.05). CON, control group; T1, group treated with *B. subtilis* KATMIRA1933; T2, group treated with *B. subtilis* KB16; T3, group treated with *B. subtilis* KB41; T4, group treated with *B. amyloliquefaciens* KB54. The data was obtained from two birds per replicated with 8 birds per group in total.

Also, there were no significant differences in most of the studied biochemical parameters. However, there were some significant changes in glucose levels among the groups (Figure 3), which do not go beyond the values described in healthy chickens (34).

### Growth Performance

There were significant differences observed in BW and ADWG among the groups during the experiment period, with the highest values in the T4 group and the least in the CON group. FCR was significantly lower in the T4 group than in the CON group in almost every studied period. It was noticed that on the 35th day of the experiment, there were no significant differences in BW, ADWG, FCR, and FI. A significant difference in FI was observed on the 42nd day in the T3 group compared to CON. While there were no significant differences in FI for the T4 group, this group showed the highest values of BW and ADWG among all groups (Table 2).

**Table 2:** Effects of *Bacillus-*feed supplementation on the growth performance of broilers during the trial.

**Period**	**Item**	**Group**					***p*-Value**	**SEM**
		**CON**	**T1**	**T2**	**T3**	**T4**		
7^th^ day	BW, g	164.2 ± 19.9^a^	165.3 ± 14.9^a^	165.5 ± 11.5^a^	169.9 ± 8.6^a, b^	175.3 ± 8.4^b^	0.0007	0.98
	ADWG, g	17.7± 2.8^a^	17.9 ± 2.1^a^	17.9 ± 1.6^a^	18.6 ± 1.2^a, b^	19.3 ± 1.2^b^	0.0007	0.14
	FCR, g/g	0.9 ± 0.1^a^	0.9 ± 0.08^a^	0.9 ± 0.07^a^	0.9 ± 0.05^a, b^	0.9 ± 0.04^a, b^	0.0004	0.01
	FI, g	151 ± 0.7	152 ± 0.8	150.5 ± 0.4	150 ± 0.9	151 ± 1.0	0.9	0.25
14^th^ day	BW, g	382.3 ± 26.7^a^	385.5 ± 46.9^a, b^	391.2 ± 37.4^a, b^	407.6 ± 34.4^b, c^	409.6 ± 21.8^b, c^	0.0006	2.55
	ADWG, g	31.2 ± 2.2^a^	31.5 ± 4.7^a, b^	32.2 ± 4^a, b^	33.9 ± 3.9^b^	33.5 ± 1.9^b^	0.0004	0.26
	FCR, g/g	1.2 ± 0.09^a^	1.2 ± 0.2^a, b^	1.2 ± 0.1^a, b^	1.1 ± 0.1^b^	1.2 ± 0.06^b^	0.001	0.01
	FI, g	471 ± 2.9	471.5 ± 3.5	471.3 ± 2.8	472 ± 3.8	472.3 ± 2.6	0.9	2.84
21^st^ day	BW, g	738.5 ± 117.2^a^	740.5 ± 108.9^a^	775 ± 98.8^a, b^	792 ± 95.6^a, b^	812.7 ± 90.1^b^	0.01	7.47
	ADWG, g	50.9 ± 13^a, b^	50.7 ± 8.9^a^	54.8 ± 8.9^a, b^	54.9 ± 8.8^a, b^	57.6 ± 10.2^b^	0.02	0.73
	FCR, g/g	1.6 ± 0.26^a^	1.6 ± 0.24^a^	1.5 ± 0.19^a, b^	1.4 ± 0.18^a, b^	1.4 ± 0.16^b^	0,01	0.02
	FI, g	1,140 ± 7.2	1,141 ± 6.2	1,141 ± 7.4	1,142 ± 5.8	1,143 ± 5.7	0.9	1.29
28^th^ day	BW, g	1,140.4 ± 122.9^a^	1,150 ± 122.9^a, b^	1,175.2 ± 112.7^a, b^	1,201.8 ± 109.5^a, b^	1,223 ± 108.2^b^	0.01	8.37
	ADWG, g	57.4 ± 1.3	58.5 ± 2.3	57.2 ± 2.9	58.6 ± 3	58.6 ± 3.4	0.009	0.19
	FCR, g/g	1.6 ± 0.18^a^	1.4 ± 0.16^b^	1.4 ± 0.14^b^	1.4 ± 0.13^b^	1.3 ± 0.12^b^	<0.0001	0.01
	FI, g	1,860 ± 21.7	1,630 ± 20.5	1,632 ± 13.6	1,634 ± 14.8	1,634 ± 15.2	0.06	20.8
35^th^ day	BW, g	1,656.5 ± 174	1,772.5 ± 174	1,697.3 ± 165.9	1,724.4 ± 157.8	1,746.4 ± 149.6	0.11	11.75
	ADWG, g	73.7 ± 8.6	74.6 ± 7.5	74.6 ± 7.7	74.7 ± 7	74.8 ± 6.1	0.98	0.52
	FCR, g/g	1.8 ± 0.19	1.7 ± 0.19	1.7 ± 0.17	1.7 ± 0.16	1.7 ± 0.15	0.16	0.01
	FI, g	2,900 ± 27.9	2,950 ± 20.9	2,920 ± 22.9	2,935 ± 20.7	2,930 ± 22.7	0.16	5.9
42^nd^ day	BW, g	2,057.9 ± 160.3^a^	2,103.5 ± 161.6^a, b^	2,127 ± 144.3^a, b^	2,155.8 ± 131.2^a, b^	2,178.4 ± 125.4^b^	0.009	10.60
	ADWG, g	57.3 ± 5.3^a^	61.6 ± 4^a^	61.4 ± 4.3^a, b^	61.6 ± 4^b^	61.7 ± 4^b^	<0.00001	0.33
	FCR, g/g	1.9 ± 0.16^a^	1.9 ± 0.15^a, b^	1.9 ± 0.13^b, c^	1.8 ± 0.11^c^	1.8 ± 0.1^c^	<0.0001	0.01
	FI, g	4,050 ± 33.2^a^	4,025 ± 27.1^a^	4,000 ± 27.9^a^	3,920 ± 21.3^b^	3,950 ± 22.1^a^	0,003	12.2

*Means within the same row without common superscripts are significantly different (p < 0.05)*.

### Isolated Gut Bacteria Composition of Broilers Treated With Potential Probiotics

Table 3 illustrates the number of microorganisms in the small intestine and cecum of the birds. Given the high variability in the number of microorganisms, the differences between the groups cannot be considered reliable. Chickens in all groups had values of bifidobacteria and lactobacilli reaching about 10^6^ CFU/g and 10^7^ CFU/g respectively.

**Table 3:** Microbiota of small intestine and cecum of broilers at 42^nd^ day.

**Microorganisms**	**Number of microorganisms (CFU/g)**							
	**Intestine**	**CON**	**T1**	**T2**	**T3**	**T4**	***p*-Value**	**SEM**
*Lactobacillus*	Small intestine	7.4 ± 0.4·10^8^	7.2 ± 0/5·10^8^	6.8 ± 0.6·10^8^	7.7 ± 0.4·10^8^	7.1 ± 0.3·10^8^	0.21	1.17
	Cecum	3.3 ± 0.5·10^8^	3.3 ± 0.5·10^8^	3.3 ± 0.5·108	3.3 ± 0.5·10^8^	3.3 ± 0.5·10^8^	0.054	3.15
*Bifidobacterium*	Small intestine	1·10^6^	1·10^6^	1·10^6^	1·10^6^	1·10^6^	1	0
	Cecum	1·10^7^	1·10^7^	1·10^7^	1·10^7^	1·10^7^	1	0
*Enterococcus*	Small intestine	2.2 ± 0.4·10^7^	2.5 ± 0.6·10^7^	2.0 ± 0.3·10^7^	1.7 ± 0.7·10^7^	1.8 ± 0.4·10^7^	0.41	1.27
	Cecum	1.5 ± 0.4·10^7^	2.4 ± 0.5·10^7^	2.1 ± 0.3·10^7^	1.2 ± 0.7·10^7^	1.6 ± 0.4·10^7^	0.053	1.47
*E.coli*	Small intestine	2.8 ± 0.3·10^7^	3.1 ± 0.3·10^7^	3.8 ± 1.1·10^7^	2.4 ± 0.9·10^7^	2.3 ± 0.8·10^7^	0.066	27.34
	Cecum	5.0 ± 1.1·10^6^	6.3 ± 0.4·10^6^	5.4 ± 0.7·10^6^	4.8 ± 1.2·10^6^	5.1 ± 0.9·10^6^	0.196	2.27
Lactose-positive bacteria	Small intestine	7.9 ± 1.3·10^5^	4.3 ± 1.2·10^5^	5.4 ± 0.9·10^5^	6.8 ± 0.7·10^5^	6.6 ± 0.7·10^5^	0.056	3.75
	Cecum	4.3 ± 1.2·10^5^	4.0 ± 1.0·10^5^	3.8 ± 0.4·10^5^	3.3 ± 0.8·10^5^	5.2 ± 1.3·10^5^	0.152	2.64
*Bacillus*	Small intestine	0	3.0 ± 0.2·10^2b^	2.6 ± 0.4·10^3b^	5.3 ± 0.5·10^3b^	4.1 ± 0.3·10^2b^	0.009	4.8
	Cecum	0	7.1 ± 0.3·10^1b^	3.9 ± 0.2·10^4b^	1.1 ± 0.1·10^4b^	3.2 ± 0.3·10^3b^	0.009	6.58

*CON, control group; T1, group treated with B. subtilis KATMIRA1933; T2, group treated with B. subtilis KB16; T3, group treated with B. subtilis KB41; T4, group treated with B. amyloliquefaciens KB54. Means within the same row without common superscripts are significantly different (p < 0.05)*.

A small number of *Bacillus* spores and vegetative cells were detected in the crop and proventriculus of the control group that did not receive probiotics. Most likely, they got there from the bedding. Already in the gizzard, they were not detected.

In the gut of group T1, trace amounts of spores were detected only in the proventriculus and gizzard. Vegetative cells were identified at very low numbers from the crop to the cecum.

In the T2 group, all bacilli cells were in a vegetative form. Their numbers increased rapidly from the small intestine, nearly reaching 10^5^ CFU/g in the colon compared to smaller amounts in the crop, proventriculus, and gizzard.

In the T3 group, vegetative cells were observed in the crop and proventriculus, reaching 1.1 × 10^4^ CFU/g. Their numbers dropped in the gizzard and then increased again, reaching a maximum of 1.1 × 10^4^ CFU/g in the cecum. The concentration of spores was up to 8.0 × 10^2^ CFU/g isolated from the cecum and colon.

**Figure 4:**
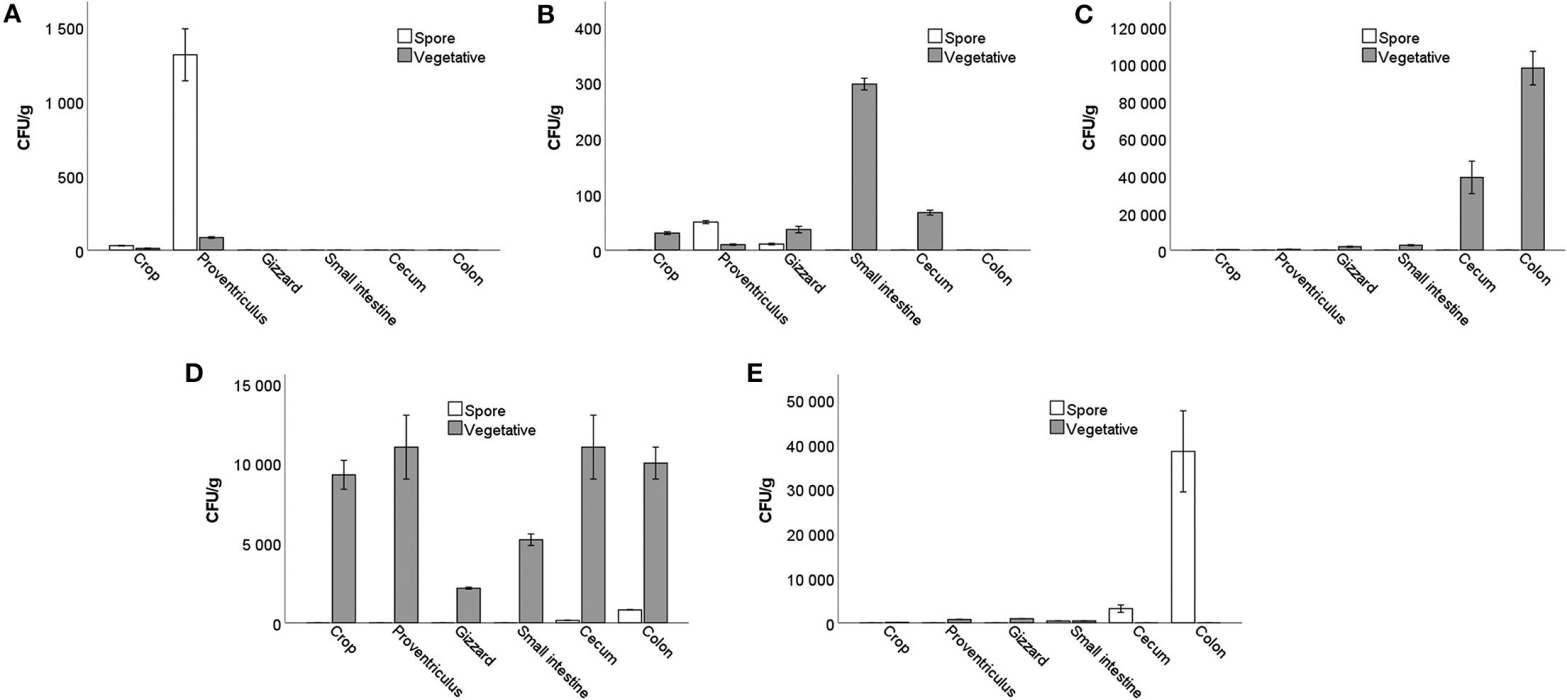
The number of *Bacillus* vegetative cells and spores in the different parts of the gastrointestinal tract in birds treated with: **(A)** basal diet without probiotic supplementation; **(B)** *B. subtilis* KATMIRA1933; **(C)** *B. subtilis* KB16; **(D)** *B. subtilis* KB41; **(E)** *B. amyloliquefaciens* KB54.

In the gut of the T4 group, vegetative cells were found in small numbers (9.0 × 10^2^ CFU/g) but were not identified in the cecum. However, *Bacillus* spores were detected in the small intestine, reaching 3.8 × 10^4^ CFU/g in the rectum (Figure 4). It can be noticed that the number of both spores and vegetative cells differs from strain to strain, although the initial number of spores obtained with food was the same for all birds.

### IL-6 and IL-10 Expression

**Figure 5:**
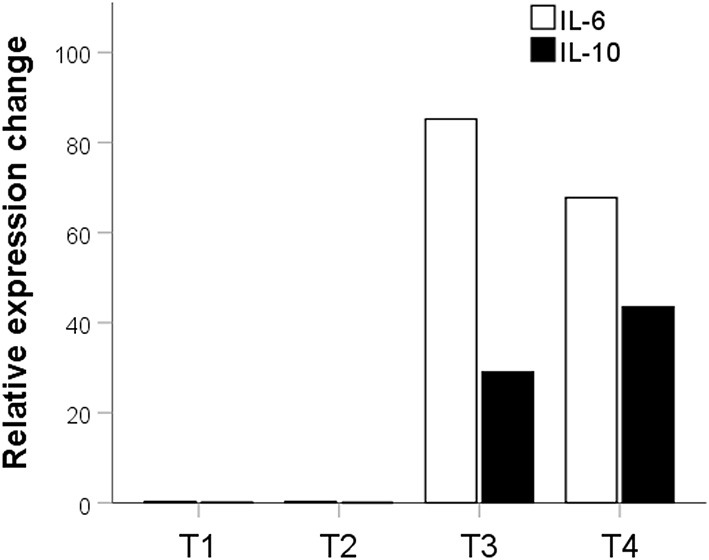
Fold expression difference of IL-6 and Il-10 in groups treated with probiotics relative to the control group. The description is given in the text. T1, group treated with *B. subtilis* KATMIRA1933; T2, group treated with *B. subtilis* KB16; T3, group treated with *B. subtilis* KB41; T4, group treated with *B. amyloliquefaciens* KB54.

Potential probiotic in the T2 group had no significant effect on the expression of IL-10 (*p* = 0.016) and IL-6 (*p* = 0.02). The potential probiotic of the T1 group also did not significantly affect the expression of IL-10 (*p* = 0.2). However, they decreased the expression of IL-6 by 3.45 times compared to the control (*p* = 0.0016). Potential probiotics from T3 and T4 groups significantly increased the expression of both pro and anti-inflammatory cytokines. In the T3 group, there was a significant increase in IL-6 expression by 85.21 times (*p* = 0.0000001) and IL-10 by 29.02 times (*p* = 0.002). There was also a significant increase in the expression of IL-6 by 67.75-fold (*p* = 0.0000001) and IL-10 - by 43.51-fold (*p* = 0.002) in the T4 group (Figure 5).

## Discussion

In this study, we investigated the effect of four *Bacillus*-based probiotic preparations on growth performance, biochemical blood parameters, and the immune system of Cobb500 broilers.

We sampled the litter from seven locations in the Rostov and Krasnodar Regions and used litter from birds of different breeds, including egg crosses and broiler chickens, as well as from different housing types, including cages, open flooring, and free-range areas. Nineteen bacilli strains were selected from the litter. All selected strains were examined for safety, antioxidant, and antimutagenic activity. For *in vivo* studies we used *B. subtilis* KB 16, *B. subtilis* KB41, and *B. amyloliquefaciens* KB54 strains, which showed the highest pooled antimutagenic and antioxidant activities *in vitro*, which was determined using lux biosensors. Lux-biosensor studies are one of the methods used for the selection of potential probiotics (23, 35, 36).

The main objective of using probiotics in livestock is to improve the animals' well-being and the quality of animal-derived products for human consumption (13). Our data reported significant differences in BW and ADWG among the groups during the whole experimental period. By day 42, a significant increase in the broilers' BW was observed in groups T3 and T4. Our findings corresponded to the other studies on the beneficial effect of *Bacillus*-based probiotics on growth performance in broilers (37–40). However, in some studies, *Bacillus*-based probiotics did not significantly affect BW, ADWG, and FCR in the tested groups (19, 20). Further studies are required to explain the variation among probiotic activity and their beneficial effects on the poultry industry. These studies should create the foundation for appropriate meta-analyses of the efficacy of the probiotic on poultry production (13).

The survival of *Bacillus* strains in the gut of chickens is of considerable fundamental interest and practical importance. In our study, the treated birds were not fed for 10 h before sacrificing, exceeding the average rate of food passage through the chicken intestines (41), which possibly allowed for the separation of bacteria that colonized the gastrointestinal tract from those obtained with food. The average number of spores in the feed was about 10^5^ CFU/g.

A small number of *Bacillus* spores and vegetative cells were detected in the crop and proventriculus of the control group that did not receive probiotics. Since the bacilli were not found in the gizzard and intestines, it is most likely that these cells were introduced to the birds from the external environment before sacrificing and did not have time to move along the gastrointestinal tract.

A low number of vegetative cells were detected in group T1, but no spores were identified in the lower intestine. We speculate that *B. subtilis* KATMIRA1933 could not colonize the intestines of birds as it was isolated from a dairy product, unlike other bacilli used in the study, which were isolated from poultry. The *B. subtilis* KATMIRA1933 cells quickly germinate from spores but are digested in the intestine and not excreted with the feces. This is consistent with our previous data (23). We suggest that potential *Bacillus* probiotics isolated from the animals' gastrointestinal tract could be more efficient than *Bacillus* probiotic candidates isolated from other environments.

We investigated the levels of gene expression for pro-inflammatory interleukin IL-6 and anti-inflammatory interleukin IL-10. In the T1 group, the level of IL-6 expression decreased slightly (Figure 4). The expression of both IL-6 and IL-10 increased significantly in T3 and T4, and IL-6 expression increased more than IL-10. Yitbarek et al. observed a similar increase in the expression of IL-6 and IL-10 when a blend of probiotic strains was used (39). Also, Sławinska et al. showed an increase in pro-inflammatory IL-6 and anti-inflammatory cytokine (IL-4) expressions when chickens were treated with synbiotics (42). Yitbarek et al. suggest that an increase in both pro-and anti-inflammatory cytokines indicates a general activation of the chickens' immune system (43).

Recent research suggests that IL-6 is an inflammatory inducer and a metabolic hormone associated with lipid, glucose, and protein metabolism as well as energy homeostasis (44). The activity of IL-6 could have an influence on the broilers' body weight and the decreased feed conversion rate, which was observed in T3 and T4 groups (Table 2). McGeachy et al. noticed an increase in IL-6 accompanied by high levels of IL-10 in some spleen cells (45), which could explain the increased level of IL-10 expression in our study.

The groups supplemented with *B. subtilis* KB41 and *B. amyloliquefaciens* KB54 showed a significant increase in BW, decreased FCR, and increased expression of IL-6 and IL-10 genes in the spleen. In the same groups, the bacilli in the intestine managed to pass through their entire life cycle from spore to spore. The connection between sporulation processes and changes in cell metabolism, an increase in the production of secondary metabolites, was previously explained (46, 47). We hypothesize that these strains can produce secondary metabolites under intestinal conditions that induce the chicken immune system and increase IL-6 and IL-10 expression levels.

Thus, IL-6 is released under the influence of the probiotic, which has, as mentioned above, pro-inflammatory and metabolic effects. The pro-inflammatory effect is offset by the opposite effect of IL-10. The metabolic action leads to an increase in the utilization of lipids and glucose and free fatty acid mobilization. This, in turn, can lead to improved feed conversion and increased growth rate and live weight of the birds, which were observed at the end of the experiment.

Similar data were obtained by Wu et al. in their study of the immune system and growth performance of chickens. The authors observed significant changes in ADWG and FI against the background of increasing serum IL-6 and IL-10 levels (48). Perhaps we can talk about a new mechanism of probiotic supplementation's effect on growth performance through the influence of bacterial metabolites on the immune system. This assumption needs further verification.

One of the study's limitations is a relatively low number of replicates per group. The effect of a relatively low number of replicates can be seen in the statistical outcomes of FI data, which are based on each replicate. The data with more variables obtained from each bird (BW, ADWG, and calculated FCR) have more significance, corresponding to basic biostatistics principles (49). This indicates that the number of replicates per each group of birds should be increased in future studies, or FI should be calculated for each bird. The most debatable limitation of our study is the BW values of Cobb500 broilers on the 42nd day of the experiment: CON – 2,057.9 g, T1 – 2,103.5 g, T2 – 2,127, T3 – 2,155.8, T4 – 2,178.4. Calik et al. reported nearly 2,500 g of Cobb500 broilers' BW in control and treatment groups on the 42nd day of their experiment (50). The mean weight gain of Cobb500 broilers on the 42nd day of the Manfio et al. study was 2,828.6–2,806.9 g depending on the experiment condition (51). Castro et al. reported BW values in the negative control group on the 42nd day that are similar to our data (2,082 g). However, BW values in other groups treated with methionine are slightly greater (>2,500 g) (52). Mutisya et al. reported a mean BW among all groups from 1,990.0 g to 2,033.9 g, which corresponds to our data (53). Also, Shawle et al. showed BW values of Cobb500 broilers on the 42nd day of the experiment that are less than our data (1,551–1,658 g) (54). All of these reports above show variations in BW data of the same Cobb500 cross broilers, which probably occurred due to uncontrolled factors. We speculate that certain compounds of the basal diet can contribute to the various values of BW, as there is no standardized nutrient content of the experimental diets in the discussed studies.

## Conclusions

Our study sheds light on the significance of some *Bacillus* probiotics used to promote growth performance in broiler chickens. *Bacillus* probiotics KB41 and KB54, isolated from chicken intestines, showed the most promising effects by improving BW, ADWG, and FCR. The leukocyte composition and biochemical blood parameters demonstrated the safety of the tested bacilli strains. Elevation of IL-6 and IL-10 expression in broilers treated with the tested *Bacillus* probiotics can open new frontiers in understanding the relationship between immune system modulation and enhancement of growth performance (BW, ADWG, and FCR).

## Data Availability Statement

The raw data supporting the conclusions of this article will be made available by the authors, without undue reservation.

## Ethics Statement

The animal study was reviewed and approved by Ethics Committee of the Don State Technical University, Rostov-on-Don, Russia.

## Author Contributions

AB, AE, AA, and MC: conceptualization and study design. IP, GZ, and VC: conducting experiments. MM: microbiota analysis. EP and AR: RNA isolation and qPCR analysis. EP and RW: lux-biosensors study and analysis. IP and AA: biostatistical analysis. MM, IP, and AR: writing—original draft preparation. MC: supervision. All authors: writing—review and editing. All authors have read and agreed to the published version of the manuscript.

## Funding

This research was funded by the Ministry of Science and Higher Education of the Russian Federation (Project No. 075-15-2019-1880).

## Conflict of Interest

The authors declare that the research was conducted in the absence of any commercial or financial relationships that could be construed as a potential conflict of interest.

## Publisher's Note

All claims expressed in this article are solely those of the authors and do not necessarily represent those of their affiliated organizations, or those of the publisher, the editors and the reviewers. Any product that may be evaluated in this article, or claim that may be made by its manufacturer, is not guaranteed or endorsed by the publisher.

## Footnotes

^1^ https://docs.cntd.ru/document/1200030168

^2^ https://docs.cntd.ru/document/1200025059
